# Diversity Patterns of Plant Communities along an Elevational Gradient in Arid and Semi-Arid Mountain Ecosystems in China

**DOI:** 10.3390/plants13202858

**Published:** 2024-10-12

**Authors:** Xinrui He, Fan Yin, Muhammad Arif, Jie Zheng, Yangyi Chen, Qianwen Geng, Xilu Ni, Changxiao Li

**Affiliations:** 1 Key Laboratory of Eco-Environments in Three Gorges Reservoir Region (Ministry of Education), Chongqing Key Laboratory of Plant Ecology and Resources Research in Three Gorges Reservoir Region, School of Life Sciences, Southwest University, Chongqing 400715, China; hxr2018@email.swu.edu.cn (X.H.); yinfan0801@email.swu.edu.cn (F.Y.); muhammadarif@swu.edu.cn (M.A.); jiezheng@email.swu.edu.cn (J.Z.); cyy372771162@email.swu.edu.cn (Y.C.); gqw0725@email.swu.edu.cn (Q.G.); 2 Biological Science Research Center, Academy for Advanced Interdisciplinary Studies, Southwest University, Chongqing 400715, China; 3 Breeding Base for State Key Laboratory of Land Degradation and Ecological Restoration in Northwest China, College of Ecology and Environment, Ningxia University, Yinchuan 750021, China; nixilu110@163.com

**Keywords:** arid and semi-arid ecosystem, biodiversity conservation, elevational gradient, vegetation classification

## Abstract

Quantitative classification and ordination are instrumental in improving our understanding of plant community patterns and facilitating effective conservation efforts in national mountain ecosystems worldwide. However, there has been a lack of relevant research focused on arid and semi-arid mountain ecosystems. This study aims to address this gap by investigating the Ningxia Helan Mountain National Nature Reserve (located in Northwest China). We conducted a comprehensive study on the patterns of plant communities and their association with environmental factors across a broad elevation range from 1200 m a.s.l. to 2600 m a.s.l. Our findings revealed the presence of 121 angiosperm species across 41 families, with vegetation classified into six distinct groups through two-way indicator species analysis (TWINSPAN) along the elevational gradient. Notably, the communities of *Ulmus*, *Prunus*, and *Stipa* in the middle elevation range exhibited the highest Shannon–Wiener (SW) and Simpson (SN) diversity indices, and these indices followed a single-peak pattern with increasing elevation. Canonical correspondence analysis (CCA) further revealed six distinct yet interrelated plant communities, revealing elevation (ELE) and the biological aridity index (BK) as the most influential environmental factors influencing plant communities’ distribution. This understanding is critically important for biodiversity conservation and the management of ecosystems in arid and semi-arid mountain ecosystems.

## 1. Introduction

The relationship between global climate change and terrestrial ecosystems is a key focus of global change research. Vegetation plays a vital role within these ecosystems, and any changes in terrestrial ecosystems will inevitably manifest as variations in vegetation types, quantities, or qualities. Hence, vegetation serves as a comprehensive indicator of ecological change [1]. Conducting regional studies on vegetation changes, along with analyzing the relationship between climate and vegetation, is crucial for understanding how climate change interacts with terrestrial ecosystems [2].

In general, mountainous regions, with their rich flora and fauna resources and highly heterogeneous habitats, are a priority area for global biodiversity conservation. Investigating the interactions between climate and ecosystems, as well as uncovering the geo-ecological principles associated with mountainous topography, will offer a scientific foundation for understanding the ecological adaptation to climate change. Mountain areas play an essential role in global biodiversity [3,4], particularly in arid and semi-arid regions where harsh environmental conditions limit overall species richness (S) [5]. Despite these challenges, mountains provide essential refuges due to their diverse topography, which fosters a range of microclimates and habitats. Studies have highlighted that the unique ecological conditions found in mountainous areas are critical for conservation efforts, as they support a wide array of life forms and endemic species [6,7]. Therefore, mountains not only serve as vital sanctuaries for biodiversity but also form an essential foundation for our understanding and protection of ecological environments. Further research into the impacts of climate change on these regions can provide clearer guidance for global biodiversity conservation efforts [8].

Species composition describes the types of species present in a specific area, and their relative proportions reflect the structure of the vegetation [9]. Environmental factors play a significant role in shaping vegetation forms and distributions [10], with climate being particularly influential in arid and semi-arid regions [11]. Plant diversity is constrained by a variety of environmental factors [12]. At smaller scales, topography and soil fertility are particularly influential, whereas at larger scales, elevation (ELE) and climate emerge as the primary determinants [13]. Despite extensive research over the years into this significant topic, debates continue regarding the key environmental factors shaping patterns of plant diversity [14]. Among the various environmental variables, ELE is pivotal in determining species diversity [15,16], and is essential for understanding changes in vegetation structure and distribution [17,18]. The substantial elevation differences found in mountainous regions lead to unique ecological and physical geographic characteristics. Leveraging elevational zonation is crucial for decoding the biogeography of mountain ecosystems [19].

Quantitative classification and ordering of plant communities can objectively and accurately reveal the complex ecological relationships between plant communities and their environment, making them essential tools in vegetation ecology [20]. Among them, quantitative classification can reveal the formation of plant communities and their relationship with environmental factors, serving as a key method to analyze the intermittency of vegetation. However, it falls short in describing the continuous distribution of communities [21]. In contrast, ordination focuses on studying vegetation continuity, thus elucidating the interrelationships between communities and their environments [22]. Since plant communities exhibit both continuity and discontinuity, a combined approach using quantitative classification and ordination is necessary to thoroughly explore the spatial distribution patterns of plant communities and their influencing factors [23]. These studies ultimately help clarify the relationship between species and their environments, providing a scientific foundation for vegetation restoration, forest management, and biodiversity conservation [24]. Different ecological schools worldwide have developed various methods and techniques for classifying vegetation communities, reflecting their distinct perspectives on composition, structure, and appearance [25]. Among these, two-way indicator species analysis (TWINSPAN) stands out as the most established and widely used quantitative classification method. It can classify samples through significant values, and can complete the classification of samples and species at the same time. Researchers have successfully applied it to quantitatively classify various types of communities, especially in community ecology studies [21,26]. Quantitative ranking methods elucidate the continuous distribution of plant communities and can quantitatively assess the distribution patterns between compositional variations and habitat factors. This contrasts with quantitative classification methods, which emphasize the intermittent distribution of communities [27].

The Ningxia Helan Mountain National Nature Reserve (China) serves as a typical example of arid and semi-arid mountain ecosystems, characterized by its vertical vegetation distribution and diverse natural environment. Thus, it is of great significance to understand the diversity patterns of plant communities along the elevational gradient of the Helan Mountain [28,29]. The objectives of this study are to (1) identify the primary vertical vegetation zones through a classification and ordination system, (2) examine the diversity differences among communities and their relationship to elevation, and (3) explore the key environmental factors influencing vegetation distribution. Based on these objectives, we hypothesize that the diversity patterns of plant communities are distinct along the elevational gradient due to variations of key environmental factors. The results of this research will provide a foundation for effective conservation planning in arid and semi-arid mountainous regions.

## 2. Results

### 2.1. Species Composition and Distribution of the Plant Communities

**Figure 1:**
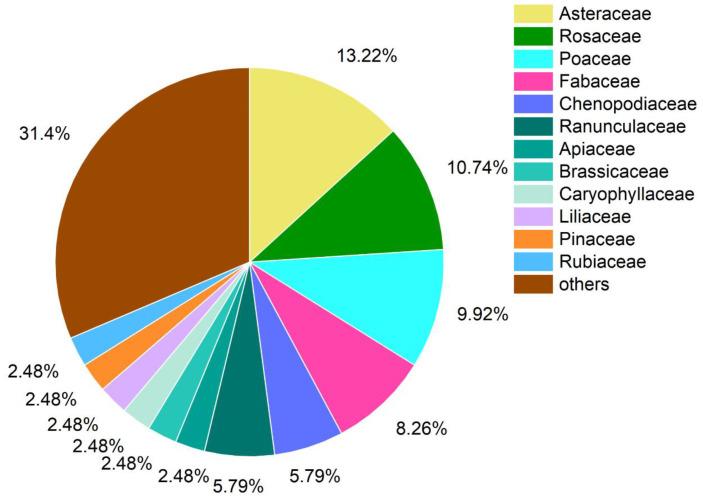
Families of the 121 plant species identified in the sampling sites.

A total of 121 plant species of angiosperms from 41 families were recorded (Table S1). The most prevalent family was Asteraceae (16 species), followed by Rosaceae (13 species), Poaceae (12 species), Fabaceae (10 species), Chenopodiaceae (7 species), and Ranunculaceae (7 species). The abovementioned families comprised 53.72% of plant species (Figure 1).

**Figure 2:**
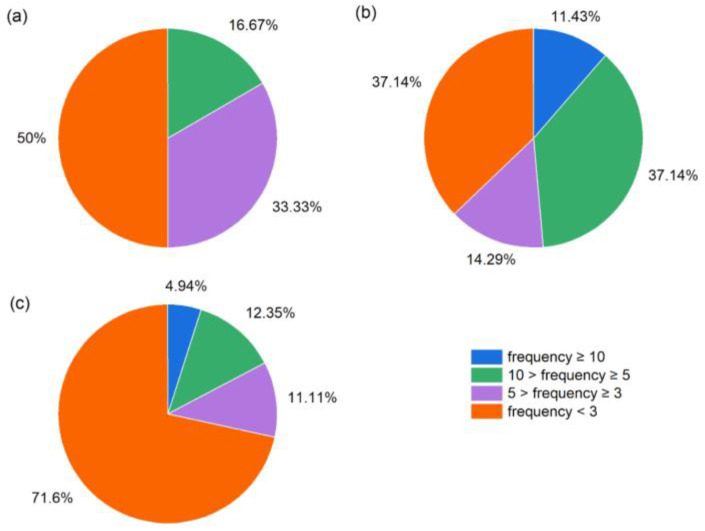
Frequency of occurrence of each species. (**a**): tree layer; (**b**): shrub layer; (**c**): herb layer.

In all surveyed sampling sites, arboreal species comprised 4.96% of the total species recorded (Table S2), with *Juniperus rigida* Siebold and Zucc. being the most frequently observed species across the sites. Additionally, three arboreal species (*Picea crassifolia* Kom., *Populus davidiana* Dode, and *Picea asperata* Mast.) were recorded at only one site, representing 50% of all species in the tree layer (Figure 2a).

Shrub-layer species were recorded, accounting for 28.93% of all species. The species with a frequency of at least ten occurrences among the sites were *Convolvulus tragacanthoides* Turcz. and *Oreosalsola laricifolia* (Litv. ex Drobow) Akhani (both 11 times), and *Prunus mongolica* Maxim. and *Dasiphora parvifolia* (Fisch. ex Lehm.) Juz. (both 10 times). A total of thirteen species were recorded in the shrub layer at only one or two sites (Table S2), comprising 10.74% of all shrub species (Figure 2b).

In the herb layer, eighty-one species were recorded, accounting for 66.94% of all species. Species occurring no less than ten times among the sites were *Stipa przewalskyi* Roshev. (13 times), *Ajania fruticulosa* (Ledeb.) Poljakov (11 times), and *Cleistogenes squarrosa* (Trin.) Keng and *Stipa tianschanica* var. *gobica* (Roshev.) P. C. Kuo and Y. H. Sun (both 10 times) (Table S2). A total of fifty-six species were found at only one or two sites, accounting for 71.60% of all species in the herb layer (Figure 2c). These results highlight considerable heterogeneity in species distribution among the sampling sites, especially in the herb layer, followed by the tree and the shrub layers, respectively (Figure 2).

### 2.2. Classification of Plant Communities and the Range of Their Environmental Variables

**Figure 3:**
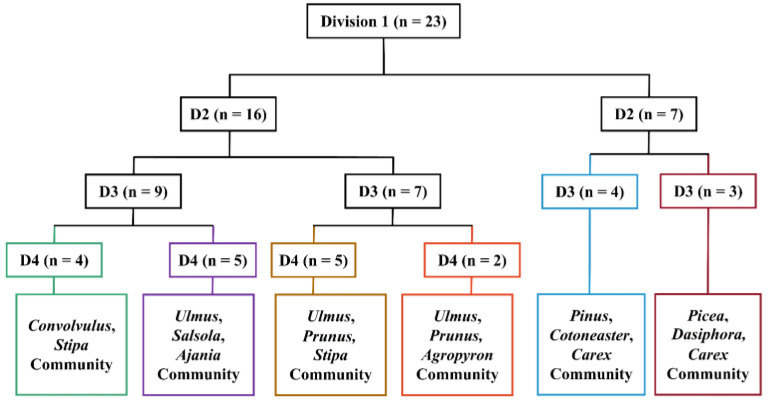
Dendrogram of the two-way indicator species analysis (TWINSPAN) classification of 23 sampling sites in the study area. “D” signifies the number of divisions, and the number in brackets denotes the count of sample plots included at each division level.

Based on the TWINSPAN classification results and their practical ecological importance, the plant communities were divided into six groups (Figure 3). The naming of communities was mainly based on the dominant species at each layer within the community.

#### 2.2.1. *Convolvulus* and *Stipa* Community (C1)

**Figure 4:**
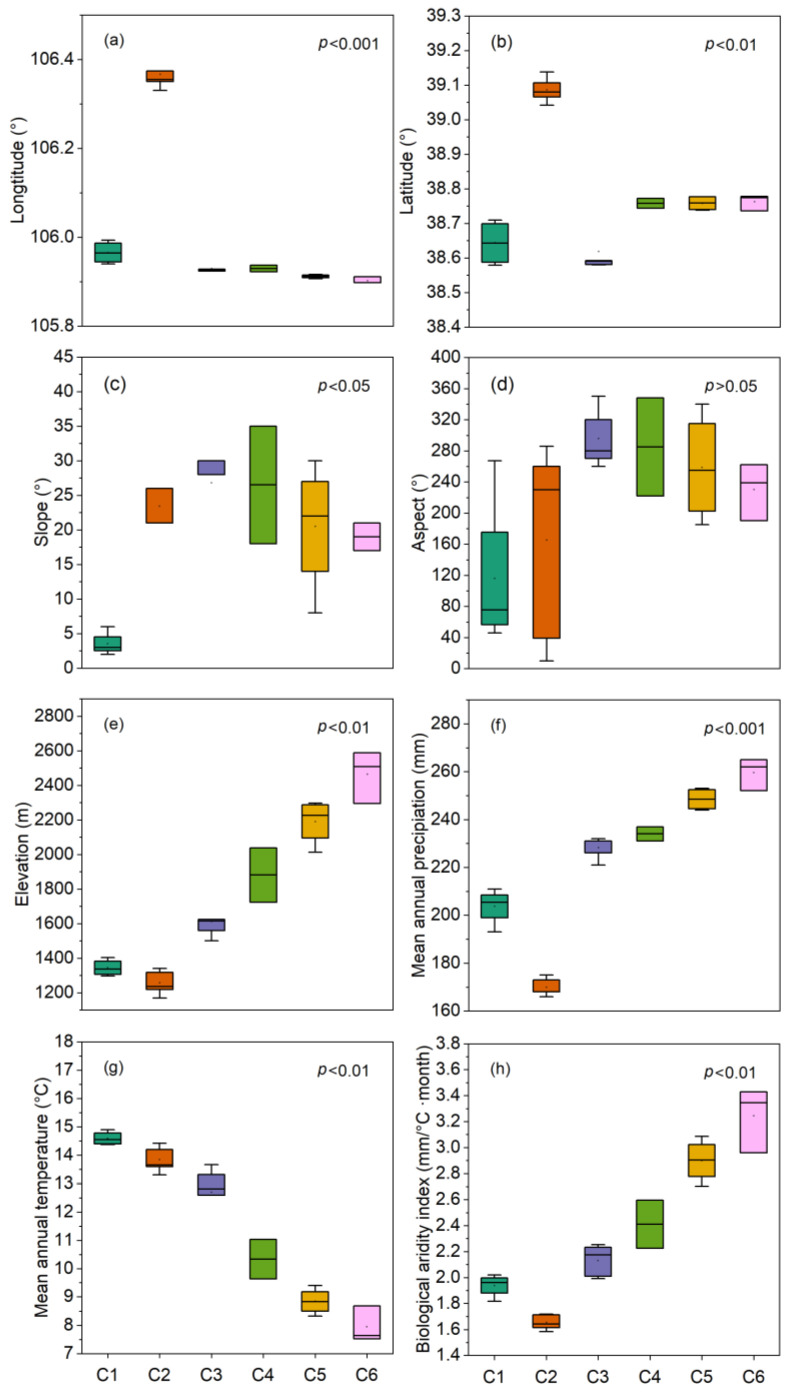
Boxplots showing the distribution ranges of environmental variables for the six plant communities classified in the study area (*p*-values indicate significant differences in means on each boxplot). C1: *Convolvulus* and *Stipa* community; C2: *Ulmus*, *Salsola*, and *Ajania* community; C3: *Ulmus*, *Prunus,* and *Stipa* community; C4: *Ulmus*, *Prunus*, and *Agropyron* community; C5: *Pinus*, *Cotoneaster*, and *Carex* community; and C6: *Picea*, *Dasiphora,* and *Carex* community. (**a**) Longitude, (**b**) Latitude, (**c**) Slope, (**d**) Aspect, (**e**) Elevation, (**f**) Mean annual precipitation, (**g**) Mean annual temperature, and (**h**) Biological aridity index).

The *Convolvulus tragacanthoides* and *Stipa tianschanica* var. *gobica* community was recorded in four sampling sites at elevations ranging from 1296 m a.s.l. to 1403 m a.s.l. (Figure 4e). A total of 24 species of plants were recorded, including 15 species in the herb layer and 9 species in the shrub layer, with no species identified in the tree layer. The common species were *Caragana stenophylla* Pojark., *Convolvulus tragacanthoides*, *Stipa przewalskyi*, and *Stipa tianschanica* var. *gobica*. The values of biological aridity index (BK) ranged from 1.82 to 2.02 (Figure 4h), with a dry and hot habitat that limits the growth of tree species (Figure 4f–h).

#### 2.2.2. *Ulmus*, *Salsola*, and *Ajania* Community (C2)

The *Ulmus glaucescens* Franch., *Salsola laricifolia*, and *Ajania fruticulosa* community was recorded at elevations between 1169 m a.s.l. and 1340 m a.s.l. (Figure 4e). A total of 32 species were recorded within this community, including 16 species in the herb layer, 15 species in the shrub layer, and 1 species in the tree layer. Common species included *Ajania fruticulosa*, *Ulmus glaucescens*, *Oxytropis aciphylla* Ledeb., *Salsola laricifolia*, and *Ptilagrostis pelliotii* (Danguy) Grubov. This community was situated in the driest habitat among the six communities studied, with a BK ranging from 1.58 to 1.72 (Figure 4h). This dryness was primarily attributed to the low mean annual precipitation (MAP), averaging only 170 mm (Figure 4f), combined with a relatively high mean annual temperature (MAT) (Figure 4g), making it the most arid habitat.

#### 2.2.3. *Ulmus*, *Prunus*, and *Stipa* Community (C3)

The *Ulmus glaucescens*, *Prunus mongolica*, and *Stipa przewalskyi* community was recorded at elevations ranging from 1500 m a.s.l. to 1768 m a.s.l. (Figure 4e). A total of 47 species were recorded within the community, including 28 herb species, 18 shrub species, and 1 tree species. Commonly found species included *Convolvulus tragacanthoides*, *Stipa przewalskyi*, *Stipa tianschanica* var. *gobica*, *Ajania fruticulosa*, *Ulmus glaucescens*, *Prunus mongolica*, and *Leptodermis ordosica* H. C. Fu and E. W. Ma. The BK for this community ranged from 1.99 to 2.25 (Figure 4h), indicating that while it had the highest number of species recorded, many of these species were present in very low abundance.

#### 2.2.4. *Ulmus*, *Prunus*, and *Agropyron* Community (C4)

The *Ulmus glaucescens*, *Prunus mongolica*, and *Agropyron mongolicum* Keng community was recorded at elevations between 1723 m a.s.l. and 2038 m a.s.l. (Figure 4e). Within this community, a total of 23 plant species were recorded, including 11 species from the herb layer, 10 species from the shrub layer, and 2 species from the tree layer. Common species included *Ulmus glaucescens*, *Prunus mongolica*, *Agropyron mongolicum*, *Rosa xanthina* Lindl., *Synotis atractylidifolia* (Y. Ling) C. Jeffrey and Y. L. Chen. The BK values for this community ranged from 2.23 to 2.60 (Figure 4h).

#### 2.2.5. *Pinus*, *Cotoneaster*, and *Carex* Community (C5)

The *Pinus tabuliformis* Carrière, *Cotoneaster zabelii* C. K. Schneid., and *Carex kansuensis* Nelmes community was recorded across elevation zones of 2013 m a.s.l. to 2296 m a.s.l. (Figure 4e). A total of 42 species were identified, including 24 herb species, 13 shrub species, and 5 tree species. Frequently observed species included *Juniperus rigida*, *Stipa przewalskyi*, *Carex kansuensis*, *Cotoneaster zabelii*, *Pinus tabuliformis*, and *Picea asperata*. The community had BK values ranging from 2.70 to 3.09 (Figure 4h).

#### 2.2.6. *Picea*, *Dasiphora*, and *Carex* Community (C6)

The *Picea crassifolia*, *Dasiphora parvifolia*, and *Carex kansuensis* community was observed across elevation zones ranging from 2294 m a.s.l. to 2589 m a.s.l. (Figure 4e). A total of 43 species were recorded, including 34 species in the herb layer, 7 species in the shrub layer, and 2 species in the tree layer. Commonly observed species included *Juniperus sabina* L., *Carex kansuensis*, *Taraxacum mongolicum* Hand.-Mazz., and *Picea crassifolia*. The value of BK for this community was the highest, with a range from 2.96–3.43 (Figure 4h), indicating that it was situated in the wettest habitat.

### 2.3. Alpha (α) Diversity Indices

**Figure 5:**
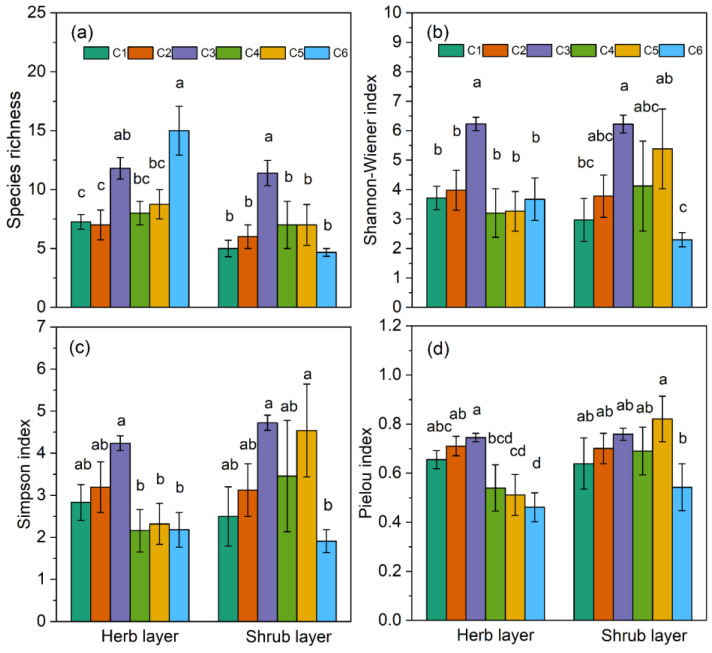
Bar charts show the differences in α-diversity of herb and shrub layers among the six plant communities classified in the study area. Different lowercase letters indicate significant differences between communities (*p* < 0.05). (**a**) Species richness, (**b**) Shannon-Wiener index, (**c**) Simpson index, and (**d**) Pielou index.

In the herb layer, the highest species richness was observed in C6, which exhibited the highest ELE, MAP and BK (Figure 4e,f,h and Figure 5a). Nevertheless, the maximum values of the other three indices, Shannon–Wiener diversity index (SW), Simpson dominance index (SN), and Pielou evenness index (Ep), were recorded in C3, except for the index Ep in the shrub layer (Figure 5b–d). In the shrub layer, C3 demonstrated significantly greater species richness compared to the other communities (Figure 5a), along with the largest mean values for SW and SN (Figure 5b,c). In contrast, in the shrub layer, all indices in C6 were lower than those in the other communities (Figure 5).

**Figure 6:**
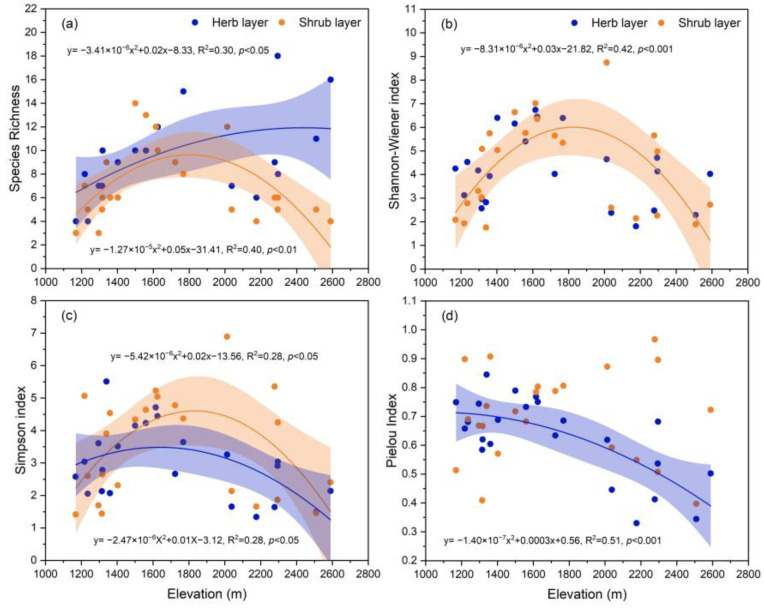
Elevational patterns of species diversity index in shrub layer and herb layer. Trend lines and shaded areas represent the fitted values from linear regression with quadratic and their 95% confidence intervals, respectively. (**a**) Species richness, (**b**) Shannon-Wiener index, (**c**) Simpson index, and (**d**) Pielou index.

Further, we investigated the species diversity indices of the shrub and herb layer across the elevational gradient (Figure 6). The results showed that species richness, SW, and SN in the shrub layer showed first an increasing and then decreasing trend (*p* < 0.05) with elevation, i.e., a unimodal distribution pattern. As for the herbal layer, SN also showed a unimodal pattern of change (*p* < 0.05), but S and Ep showed first an increasing and then decreasing trend with elevation (*p* < 0.05).

### 2.4. Ordination

**Figure 7:**
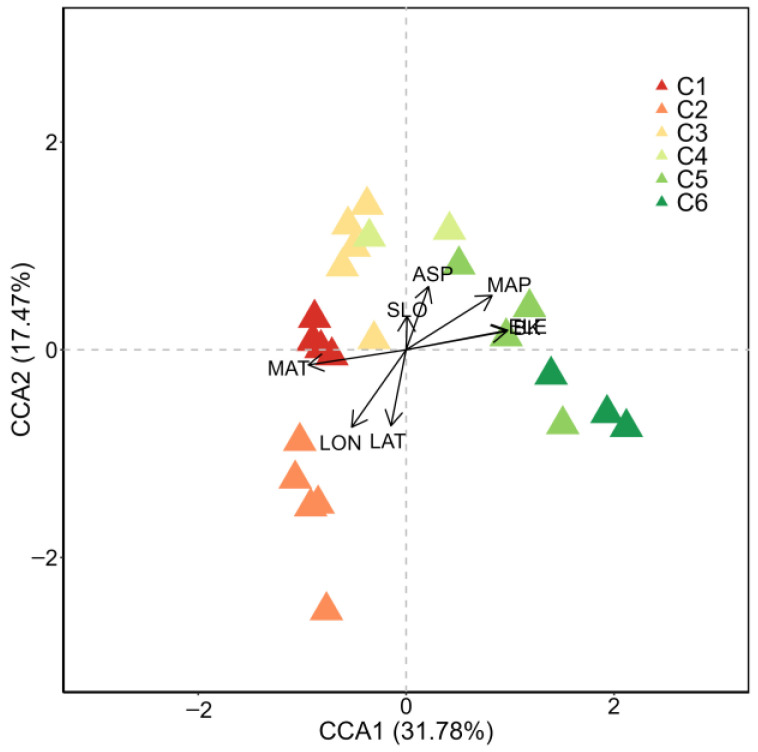
Canonical correspondence analysis (CCA) ordination plots show the relationship between community structure and environmental variables for each sampling site. The values in the labels of the axes in the figure represent the proportion of variation in species communities explained by the combination of environmental factors represented by the axes. ELE: elevation, SLO: slope, ASP: aspect, LON: longitude, LAT: latitude, MAP: mean annual precipitation, MAT: mean annual temperature, and BK: biological aridity index.

The results of the canonical correspondence analysis (CCA) showed that the environmental variables, including ELE, slope (SLO), aspect (ASP), longitude (LON), latitude (LAT), MAP, MAT, and BK, accounted for 49.25% of the variation (Figure 7). The Monte Carlo tests for CCA showed that ELE, ASP, LON, LAT, MAP, MAT, and BK were significantly related to vegetation distribution patterns (*p* < 0.01), with ELE being the most critical factor (Table 1; Figure 7).

According to the coefficient of determination (R^2^) of each environmental variable (Table 1), ELE was the most influential factor, followed by BK, and then by MAP, MAT and LON. C1, C2, and C3 were grouped on the lower elevation side, characterized by higher MAT but lower MAP and BK (Figure 4). In contrast, C4, C5, and C6 were found at higher elevations, exhibiting higher MAP and lower MAT, along with an elevated BK (Figure 4).

## 3. Discussion

### 3.1. Plant Community Characteristics

By referencing relevant methods of nomenclature and species delimitation [30,31], along with the assistance of Flora of China (http://www.iplant.cn/frps, accessed on 13 September 2023). Our results showed that among the sampling sites we examined, the family Asteraceae was the most prevalent, featuring 11 genera and 16 species, followed by Rosaceae (13 species), Poaceae (12 species), and Fabaceae (10 species) (Table S1; Figure 1), which was similar to the results of previous studies [32,33]. Asteraceae plants tend to accumulate significant amounts of fructans, which can notably enhance their resistance to drought stress [34,35]. Additionally, the symbiotic relationship between Fabaceae and Rhizobia promotes the rapid growth of Fabaceae in relatively arid conditions [36], allowing them to flourish in the reserve’s dry and low-rainfall environment. The results of the survey also showed that the number of species in different layers varied greatly (Table S2; Figure 2). This disparity arises because trees require more water for growth compared to shrubs and herbs; however, the reserve is located in an arid and semi-arid region characterized by low average annual rainfall and highly uneven intra-annual distribution, which leads to many tree species being unable to survive due to drought filtering. In contrast, *Juniperus rigida* and *Ulmus glaucescens* were found to have a higher frequency of occurrence in the reserve, as they are drought-resistant and tolerant of poor soil conditions, with a well-developed root systems that enables them to thrive in adverse environments [37]. The most commonly observed species in the shrub and herb layers were also drought-tolerant, including *Salsola laricifolia*, *Convolvulus tragacanthoides*, *Prunus mongolica*, and *Dasiphora parvifolia*, as well as *Stipa przewalskyi*, *Ajania fruticulosa*, *Cleistogenes squarrosa*, and *Stipa tianschanica* var. *gobica* (Table S2). This drought tolerance significantly contributed to their high occurrence rates within the survey area of the reserve.

### 3.2. Spatial Distribution Patterns of α-Diversity

The development of plant diversity patterns is a complex ecological process influenced by various factors, including climate, topography, and soil characteristics, all of which are interconnected and shaped by different environmental elements [14]. In our study, analysis of the α-diversity within shrub layers across various communities revealed that S, SW, and SN were highest in C3 (Figure 5a–c). In the herb layer, SW, SN, and Ep were also highest in C3 (Figure 5b–d), which supported our hypothesis. The elevational gradient encompasses the combined effects of various environmental factors such as temperature, precipitation, and solar radiation, playing a crucial role in determining the vertical distribution patterns of mountain species diversity. The most common pattern observed is a negative correlation between diversity and elevation, where species diversity declines as elevation increases [37]. Another observed pattern is unimodal, where species diversity first increases and then decreases with elevation [38,39,40], particularly prevalent in semi-arid regions. In the low elevation of arid mountain regions, the climate is characterized by year-round drought and low rainfall, resulting in relatively poor soil conditions. The growth and development of plants are typically limited by precipitation [41], and these areas are more susceptible to human disturbances, leading to lower species diversity [42]. In contrast, as high-elevation areas experience cold climates, abundant rainfall, and higher wind speeds, with less human interference, plant growth is primarily limited by temperature [43]. Compared to high elevation, middle elevation may offer a relatively favorable allocation of water and thermal resources for plant growth, resulting in greater resource utilization and species diversity [44]. This thus explains the variation in shrub layer species diversity observed in this study (Figure 6).

Climate change poses a significant threat to species diversity, largely driven by rising temperatures [45]. As the climate warms, individual species tend to shift from high-temperature areas to low-temperature areas [46]. However, different plant functional groups respond to climate change in varied ways; for instance, species with shorter life cycles and more frequent regeneration tend to shift faster [47]. The migration of low-altitude plants to high altitudes can increase the diversity of plants [46]. Meanwhile, at high elevation, species reduce competition through niche differentiation, thereby promoting the richness of the herb layer [48]. Additionally, research has indicated that arid mountain plants are particularly sensitive to changes in precipitation, allowing them to respond rapidly to the fluctuation of precipitation [49]. Higher soil moisture at high elevations can facilitate the growth of more wet demanding species, such as *Stipa przewalskyi* Roshev. and *Stipa tianschanica* var. *gobica* (Roshev.) P. C. Kuo and Y. H. Sun, and this well explains why there is a greater species richness of herbs at high elevations in the study areas (Figure 6a). However, despite the increase in species richness, limited resources intensify competition, which may lead to an increase in the abundance of certain dominant species while reducing the numbers of others [50,51], consequently lowering Ep (Figure 6d). Moreover, the extreme environmental conditions in high elevation regions (such as low temperatures and strong winds) may restrict the survival of certain species, allowing some species to dominate in these areas, and thus further exacerbating the decline in the value of Ep (Figure 6). Species diversity is influenced by a complex interplay of soil temperature and moisture. As global climate warming continues, it is essential to consider environmental factors to predict plant diversity. Additionally, research should also prioritize understanding the migration abilities of various species.

### 3.3. Classification and Ordination

Vegetation serves as a comprehensive indicator of environmental characteristics, with specific plant communities reflecting their habitat gradients. The quantitative classification of these communities is a crucial approach for studying their composition and spatial distribution patterns [52]. In our research, 23 sampling sites within the study area were categorized into six distinct communities using TWINSPAN (Figure 3), which supported our hypothesis. The ecological interpretation of the detrended correspondence analysis (DCA) ordering axes varies among researchers. Some suggest that the first axis represents a moisture gradient, while the second axis reflects a heat gradient [53]. Others argue that the first axis indicates a heat gradient (elevation) and the second axis denotes a water gradient [54]. Additionally, some scholars propose that the first axis reflects changes in community elevation, with the second axis representing variations in slope or slope direction [55,56]. A synthesis of these studies indicates that the ecological interpretation of DCA axes can lead to different conclusions depending on the study location, subjects, and scales. In regions with higher precipitation, moisture is relatively abundant, and thus, it may not limit vegetation growth; in such cases, heat may have a more significant influence on species composition and spatial distribution. Conversely, in more arid areas, moisture conditions are likely to play a crucial role in shaping community structure and vegetation distribution patterns. In terms of the habitat conditions of the communities in our study (Figure 4), there were significant differences in ELE, MAP, MAT, and BK among the communities, which also supported our hypothesis and highlighted the gradient variations in habitat conditions. The TWINSPAN classification results illustrated the discontinuity of vegetation, while the CCA indicated that some communities were clustered together yet not completely isolated, suggesting a degree of continuity along the environmental gradient. Consequently, our findings indicate that vegetation exhibits both discontinuous and continuous characteristics, and it is essential to consider both aspects when conducting field investigations on vegetation.

Plant communities are the result of intricate interactions and mutual adaptations between organisms and their environment over extended periods. Topography plays a crucial role in shaping species composition and distribution [57]. Numerous studies have identified elevation as the primary factor influencing the distribution patterns of plant communities in mountainous regions [58]. In fact, ELE is a multifaceted variable, and its variation affects environmental factors such as moisture and temperature [59,60]. It can change the species composition of the community by regulating the local water and heat distribution [61], which subsequently influences community distribution patterns. As a key topographic factor in mountain ecosystems, ASP affects habitat conditions such as soil temperature, water availability, and nutrient distribution. This influence subsequently impacts the redistribution of matter and energy, ultimately leading to changes in the composition and distribution of forest community species [62,63,64]. Additionally, precipitation is often regarded as a key factor influencing community distribution in arid and semi-arid regions [65]. Our findings indicate that MAP significantly affects vegetation distribution patterns; however, BK has an even greater influence (*p* < 0.001; Table 1; Figure 7). This could be due to BK representing a combination of temperature and precipitation, thereby providing a better insight into habitat moisture levels. Wetness and aridity index can be applied effectively to analyze the relationship between vegetation distribution and moisture and temperature factors in arid and semi-arid areas. The present study found that both ELE and BK were important factors affecting vegetation distribution. These results could further enrich our understanding regarding the relationship between vascular plants communities and environmental factors. The environmental factors analyzed accounted for only 49.43% of the variance (Figure 7). In fact, lower environmental interpretations were more common in community analyses and were present in both larger and smaller scale study areas [66]. This was mainly limited by spatial factors related to the low spatial scales of climate indicators, stochastic processes between communities and their environments, and mismatches between the spatial scales of environmental factors and community composition [27]. Future research should be carried out in these areas, for instance, a phytosociological investigation, for attributing the six detected plant communities to proper syntaxa. In fact, phytosociological surveyance and classification give some fundamental information on the synecology of plant communities [67,68], which can be used for a better conservation plan in relation to the studied ecosystems.

## 4. Materials and Methods

### 4.1. Study Area

**Figure 8:**
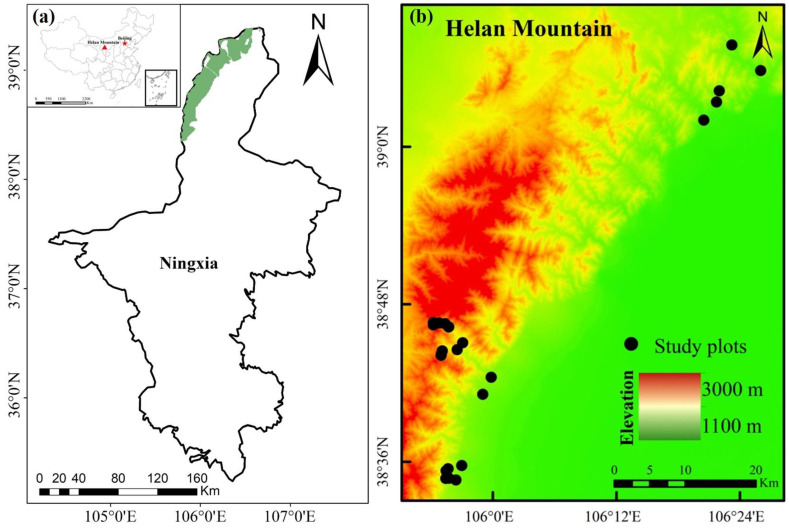
A sketch map of the study area and sampling sites. (**a**) Location of the study area in China, and (**b**) distribution of sample plots in the study area.

The study area (38°19′–39°22′ N, 105°49′–106°41′ E) was situated in the northwest region of Ningxia, China (Figure 8). In this region, the MAT fluctuates between 8.2 °C and 8.6 °C, with extremes of −8.54 °C in January and 21.43 °C in August. The MAP registers at 209.2 ± 57.2 mm, with around 44% of this precipitation occurring in July and August [32]. Typically found in temperate mountain forest ecosystems, higher elevations tend to have lower temperatures, while precipitation tends to increase with elevation. The main plant species are *Agropyron mongolicum*, *Prunus mongolica*, *Caragana stenophylla*, *Ulmus glaucescens*, *Juniperus rigida*, *Pinus tabuliformis*, and *Picea crassifolia* [69,70].

### 4.2. Field Investigation

A comprehensive investigation of the vegetation was conducted during the summer of 2021. Covering elevations ranging from 1200 m a.s.l. to 2600 m a.s.l., 23 sampling sites were selected based on site conditions along with elevation gradients of the Helan Mountains [70,71]. At each site, a 20 m × 20 m sample plot was established [72]; within each plot, one quadrat of 400 m^2^ (20 m × 20 m), five quadrats of 25 m^2^ (5 m × 5 m), and five quadrats of 1 m^2^ (1 m × 1 m) were sampled to document data on trees, shrubs, and herbs, respectively.

### 4.3. Environmental Variables

The research incorporated various environmental variables, which were divided into three categories: topographic, spatial, and climatic factors. Topographic variables encompassed SLO, ASP, and ELE. Spatial variables were represented by LAT and LON. Climate data were obtained from the global climate database WorldClim [73]. Temperature and precipitation data for each sampling site were extracted with the help of ArcGIS 10.8, which included MAP and MAT. Additionally, the BK was developed as a useful metric for evaluating the ecological moisture conditions. The value was calculated with *BK*_1_ when *BWI* ≤ 80 or *BK*_2_ when *BWI* > 80 [74]:

(1) $${BK}_{1}=\frac{APP}{BWI+40}$$

(2) $${BK}_{2}=\frac{2\times APP}{BWI+120}$$

(3) $$BWI=\sum_{i=1}^{n}{(T}_{i}-10)$$

where “*APP*” is the annual precipitation (mm); “*n*” is the number of months with the mean monthly temperature above 10 °C; “*T_i_*” is the mean monthly temperature above 10 °C.

### 4.4. Statistical Analysis

#### 4.4.1. Two-Way Indicator Species Analysis (TWINSPAN)

In our research, we employed the importance value of species to identify the prominent plant communities within the designated area, calculated using the following equations [75]:

Importance value of trees:

(4) $${IV}_{tr}=relative\;abundance+relative\;frequency+relative\;dominance$$

Importance values of shrubs and herbs:

(5) $${IV}_{sh,\;he}=relative\;abundance+relative\;frequency+relative\;coverage$$

Utilizing this importance value as the foundation, a matrix of importance values for species and samples was generated for TWINSPAN delineation [76]. To enhance the stability of subsequent analyses and reduce the impact of rare species on the classification process, we synthesized existing studies and excluded species that appeared in one plot or fewer and had a total importance value of less than 1 [77]. Finally, we opted to focus solely on the importance values of 47 species; this refined approach aims to enhance the precision of our results by prioritizing the contribution of more prevalent species to the classification process.

#### 4.4.2. α-Diversity Metrics

The α-diversity measures chosen for this study include the *S*, *SW*, *SN*, and *E_p_* [75].

(6) $$S=\sum_{i=1}^{n}N$$

(7) $$SW=-\sum_{i=1}^{n}\frac{N_{i}}{N}ln\frac{N_{i}}{N}$$

(8) $$SN=1-\sum_{i=1}^{n}{\left(\frac{N_{i}}{N}\right)}^{2}$$

(9) $$E_{p}=\frac{SW}{\ln\left(S\right)}$$

where *N_i_* is the number of individuals in species *i; N* is the total number of individuals in all species; and *n* is the number of species.

One-way ANOVA and Duncan’s multiple comparisons (*p* < 0.05) were performed to identify any significant differences in α-diversity among communities across different layers. Before using Duncan’s method, we ensured that the data met the basic assumptions of ANOVA, such as normality and homogeneity of variance test. Regression analysis was conducted to see the influence of altitude on species diversity indices using Origin 2021 [32]. In investigating the relationship between vegetation and environmental factors, the analysis began with DCA applied to the species importance value-sample matrix. Suitable sorting methods were then chosen based on the maximum values of the DCA sorting axes. Following this, the forward-selection method and a Monte Carlo permutation test (simulated 999 times) were employed to evaluate the overall significance of all environmental factors in explaining species distribution [77]. Furthermore, Z-score standardization of species significance values and environmental data was performed using the decostand function from the vegan package, resulting in the generation of a final two-dimensional ordination plot depicting the relationship between communities and environmental factors [78]. TWINSPAN classification was carried out using WinTWINS 2.3, while other statistical analyses, including DCA and CCA, were performed in R 4.3.2 using the vegan package [79,80].

## 5. Conclusions

Ningxia Helan Mountain National Nature Reserve (located in Northwest China) boasts rich plant diversity and a distinct vertical vegetation zone. Especially, the central elevation area of the reserve hosts a diverse array of plants. The vegetation was classified into six communities, namely the *Ulmus*, *Salsola*, and *Ajania* community; *Convolvulus* and *Stipa* community; *Ulmus*, *Prunus*, and *Stipa* community; *Ulmus*, *Prunus*, and *Agropyron* community; *Pinus*, *Cotoneaster*, and *Carex* community; and *Picea*, *Dasiphora*, and *Carex* community, delineated along the elevational gradient. Communities in the middle elevation range displayed the highest levels of Shannon–Wiener and Simpson indices. Additionally, their value followed a single-peak pattern with increasing elevation, particularly notable for the shrub layer. The canonical correspondence analysis results underscore elevation and biological aridity index as pivotal factors influencing vegetation distribution. The findings of this study not only enhance our comprehension of community spatial distribution patterns but also provide practical implications for protecting rare species and establishing a theoretical basis for the future management, utilization, and conservation of the reserve.

## Figures and Tables

**Table 1:** Significance test of environmental variables in the study area.

Environmental Variables	CCA1	CCA2	R^2^	*Significance*
ELE	0.99	0.17	0.96	***
SLO	0.02	0.99	0.09	ns
ASP	0.35	0.94	0.38	**
LON	−0.60	−0.80	0.76	***
LAT	−0.21	−0.98	0.50	**
MAP	0.86	0.50	0.91	***
MAT	−0.99	−0.14	0.88	***
BK	0.99	0.17	0.94	***

Each combination indicated significant differences at *p* < 0.001 (***) and *p* < 0.01 (**) respectively. ELE: elevation, SLO: slope, ASP: aspect, LON: longitude, LAT: latitude, MAP: mean annual precipitation, MAT: mean annual temperature, and BK: biological aridity index.

## Data Availability

Data are contained within the article and Supplementary Materials.

## Supplementary Materials

The following supporting information can be downloaded at: https://www.mdpi.com/article/10.3390/plants13202858/s1, Table S1: Plant species identified from the study area.; Table S2: Frequency statistics of the occurrence of each species among the 23 sampling sites.

## References

[1] Parmesan C., Yohe G. (2003). A globally coherent fingerprint of climate change impacts across natural systems. Nature.

[2] Fu B.J., Niu D., Zhao S.D. (2005). Study on global change and terrestrial ecosystems: History and prospect. Adv. Earth Sci..

[3] Garcia-Porta J., Simo-Riudalbas M., Robinson M., Carranza S. (2017). Diversification in arid mountains: Biogeography and cryptic diversity of *Pristurus rupestris rupestris* in Arabia. J. Biogeogr..

[4] Rahbek C., Borregaard M.K., Colwell R.K., Dalsgaard B., Holt B., Morueta-Holme N., Nogués-Bravo D., Whittaker J., Fjeldså J. (2019). Humboldt’s enigma: What causes global patterns of mountain biodiversity?. Science.

[5] Shi H., Tian H.Q., Lange S., Yang J., Pan S.F., Fu B.J., Christopher P.O. (2021). Terrestrial biodiversity threatened by increasing global aridity velocity under high-level warming. Proc. Natl. Acad. Sci. USA.

[6] Thomas J., El-Sheikh M.A., Alatar A.A. (2017). Endemics and endangered species in the biodiversity hotspot of the Shada Mountains, Saudi Arabia. J. Arid Land.

[7] Di Pietro R., Wagensommer R.P. (2014). A new Sesleria juncifolia association from south-eastern Italy and its position in the amphi-Adriatic biogeographical context. Acta Bot. Croat..

[8] Payne D., Spehn E.M., Snethlage M., Fischer M. (2017). Opportunities for research on mountain biodiversity under global change. Curr. Opin. Environ. Sustain..

[9] Sheridan J.A., Kendrick M.R. (2024). Relationships of primary productivity with anuran abundance, richness, and community composition in tropical streams. PLoS ONE.

[10] Rahman I.U., Afzal A., Iqbal Z., Bussmann R.W., Alsamadany H., Calixto E.S., Shah G.M., Kausar R., Shah M., Ali N. (2020). Ecological gradients hosting plant communities in Himalayan subalpine pastures: Application of multivariate approaches to identify indicator species. Ecol. Inform..

[11] Castillón E.E., Arévalo J.R., Quintanilla J.A.V., Rodríguez M.M.S., Encina-Domínguez J.A., Rodríguez H.G., Ayala C.M.C. (2015). Classification and ordination of main plant communities along an altitudinal gradient in the arid and temperate climates of northeastern Mexico. Sci. Nat..

[12] Liang H.Z., Fu T.G., Gao H., Li M., Liu J.T. (2023). Climatic and non-climatic drivers of plant diversity along an altitudinal gradient in the Taihang Mountains of northern China. Diversity.

[13] Zu K.L., Luo A., Shrestha N., Liu B., Wang Z.H., Zhu X.Y. (2019). Altitudinal biodiversity patterns of seed plants along Gongga Mountain in the southeastern Qinghai-Tibetan Plateau. Ecol. Evol..

[14] Fan Z.M., Bai R.Y., Yue T.X. (2019). Spatio-temporal distribution of vascular plant species abundance on Qinghai-Tibet Plateau. J. Geogr. Sci..

[15] Shooner S., Davies T.J., Saikia P., Deka J., Bharali S., Tripathi O.P., Singha L., Khan M.L., Dayanandan S. (2018). Phylogenetic diversity patterns in Himalayan forests reveal evidence for environmental filtering of distinct lineages. Ecosphere.

[16] Tolmos M.L., Kreft H., Ramirez J., Ospina R., Craven D. (2022). Water and energy availability mediate biodiversity patterns along an elevational gradient in the tropical Andes. J. Biogeogr..

[17] Worthy S.J., Paz R.A.J., Pérez A.J., Reynolds A., Cruse-Sanders J., Valencia R., Barone J.A., Burgess K.S. (2019). Distribution and community assembly of trees along an Andean elevational gradient. Plants.

[18] Song X.Y., Cao M., Li J.Q., Kitching R.L., Nakamura A., Laidlaw M.J., Tang Y., Sun Z.H., Zhang W.F., Yang J. (2021). Different environmental factors drive tree species diversity along elevation gradients in three climatic zones in Yunnan, southern China. Plant Divers..

[19] Fattorini S., Di Biase L., Chiarucci A. (2019). Recognizing and interpreting vegetational belts: New wine in the old bottles of a von Humboldt’s legacy. J. Biogeogr..

[20] Burke A. (2001). Classification and ordination of plant communities of the Naukluft mountains, Namibia. J. Veg. Sci..

[21] Bai X.H., Zhang J.T., Cao K., Wang Y.Q., Sadia S., Cao G. (2017). Relationship between forest communities and the environment in the Xiaowutai Mountain National Nature Reserve, Hebei. Acta Ecol. Sin..

[22] Virtanen R., Luoto M., Rämä T., Mikkola K., Hjort J., Grytnes J.A., Birks H.J.B. (2010). Recent vegetation changes at the high-latitude tree line ecotone are controlled by geomorphological disturbance, productivity and diversity. Glob. Ecol. Biogeogr..

[23] Li T.T., Ji L.Z., Yu D.P., Zhou L., Zhou W.M., Mao Y.X., Dai L.M. (2019). Forest community classification, ordination, and comparison of species diversity in broadleaved-Korean pine mixed forests of Northeast China. Acta Ecol. Sin..

[24] Zhao H., Wang Q.R., Fan W., Song G.H. (2017). The relationship between secondary forest and environmental factors in the Southern Taihang mountains. Sci. Rep..

[25] Whittaker R.H. (1978). Classification of Plant Communities.

[26] Liu Y.Z., Shen H.H., Ge G., Xing A.J., Tang Z.Y., Fang J.Y. (2023). Classification and distribution of evergreen broad-leaved forests in Jiangxi, East China. J. Plant Ecol..

[27] Bao X.T., Ding L.B., Yao S.C., Wang J.S., Shi P.L., Wang T., Li C., Liu W.J. (2019). Quantitative classification and ordination of grassland communities on the Lhasa River Basin. Acta Ecol. Sin..

[28] Guo K., Liu C.C., Xie Z.Q., Li F.Y., Franklin S.B., Lu Z.J., Ma K.P. (2018). China Vegetation Classification: Concept, approach and applications. Phytocoenologia.

[29] Wu P.P., Wang Z., Jia N.X., Dong S.Q., Qu X.Y., Qiao X.G., Liu C.C., Guo K. (2022). Vegetation classification and distribution patterns in the south slope of Yarlung Zangbo Grand Canyon National Nature Reserve, Eastern Himalayas. Plants.

[30] Turland N.J., Wiersema J.H., Barrie F.R., Greuter W., Hawksworth D.L., Herendeen P.S., Knapp S., Kusber W.H., Li D.Z., Marhold K. (2018). International Code of Nomenclature for Algae, Fungi, and Plants (Shenzhen Code).

[31] Rouhan G., Gaudeul M. (2021). Plant Taxonomy: A Historical Perspective, Current Challenges, and Perspectives. Methods Mol. Biol..

[32] Zheng J., Arif M., He X.R., Ding D.D., Zhang S.L., Ni X.L., Li C.X. (2022). Plant community assembly is jointly shaped by environmental and dispersal filtering along elevation gradients in a semiarid area, China. Front. Plant Sci..

[33] Geng Q.W., Arif M., Yuan Z.X., Zheng J., He X.R., Ding D.D., Yin F., Li C.X. (2022). Plant species composition and diversity along successional gradients in arid and semi-arid regions of China. For. Ecol. Manag..

[34] Bernard L., Decau M.L., Morvan-Bertrand A., Lavorel S., Clément J.C. (2020). Water-soluble carbohydrates in *Patzkea paniculata* (L.): A plant strategy to tolerate snowpack reduction and spring drought in subalpine grasslands. Plant Biol..

[35] Rigui A.P., Carvalho V., dos Santos A.L.W., Morvan-Bertrand A., Prud’homme M.P., de Carvalho M.A.M., Gaspar M. (2019). Fructan and antioxidant metabolisms in plants of *Lolium perenne* under drought are modulated by exogenous nitric oxide. Plant Physiol. Biochem..

[36] Igiehon N.O., Babalola O.O., Aremu B.R. (2019). Genomic insights into plant growth promoting rhizobia capable of enhancing soybean germination under drought stress. BMC Microbiol..

[37] Marathe A., Priyadarsanan D.R., Krishnaswamy J., Shanker K. (2020). Spatial and climatic variables independently drive elevational gradients in ant species richness in the Eastern Himalaya. PLoS ONE.

[38] Sun L., Luo J., Qian L.S., Deng T., Sun H. (2020). The relationship between elevation and seed-plant species richness in the Mt. Namjagbarwa region (Eastern Himalayas) and its underlying determinants. Glob. Ecol. Conserv..

[39] Xu M.H., Zhang S.X., Wen J., Yang X.Y. (2019). Multiscale spatial patterns of species diversity and biomass together with their correlations along geographical gradients in subalpine meadows. PLoS ONE.

[40] Xu M.H., Du R., Li X.L., Yang X.H., Zhang B.G., Yu X.L. (2021). The mid-domain effect of mountainous plants is determined by community life form and family flora on the Loess Plateau of China. Sci. Rep..

[41] Wang Z.H., Tang Z.Y., Fang J.Y. (2007). Altitudinal patterns of seed plant richness in the Gaoligong Mountains, south-east Tibet, China. Divers. Distrib..

[42] da Silva F.K.G., de Faria Lopes S., Lopez L.C.S., de Melo J.I.M., Trovão D.M.d.B.M. (2014). Patterns of species richness and conservation in the Caatinga along elevational gradients in a semi arid ecosystem. J. Arid. Environ..

[43] Tang Z.Y., Fang J.Y. (2004). A review on the elevational patterns of plant species diversity. Biodivers. Sci..

[44] McCain C.M. (2007). Could temperature and water availability drive elevational species richness patterns? A global case study for bats. Glob. Ecol. Biogeogr..

[45] Sedlacek J., Wheeler J.A., Cortés A.J., Bossdorf O., Hoch G., Lexer C., Wipf J., Karrenberg S., Kleunen M.V., Rixen C. (2015). The response of the alpine dwarf shrub Salix herbacea to altered snowmelt timing: Lessons from a multisite transplant experiment. PLoS ONE.

[46] Steinbauer M.J., Grytnes J.A., Jurasinski G., Kulonen A., Lenoir J., Pauli H., Rixen C., Winkler M., Bardy-Durchhalter M., Barni E. (2018). Accelerated increase in plant species richness on mountain summits is linked to warming. Nature.

[47] Wheeler J.A., Schnider F., Sedlacek J., Cortés A.J., Wipf S., Hoch G., Rixen C. (2015). With a little help from my friends: Community facilitation increases performance in the dwarf shrub Salix herbacea. Basic Appl. Ecol..

[48] Wang C.T., Long R., Wang Q.J., Ding L.M., Wang M.P. (2007). Effects of altitude on plant-species diversity and productivity in an alpine meadow, Qinghai-Tibetan plateau. Aust. J. Bot..

[49] Du J., Li K., He Z.B., Chen L.F., Lin P.F., Zhu X. (2020). Daily minimum temperature and precipitation control on spring phenology in aridmountain ecosystems in China. Int. J. Climatol..

[50] Niu Y.J., Yang S.W., Zhou J.W., Chu B., Ma S.J., Zhu H.M., Hua L.M. (2018). Vegetation distribution along mountain environmental gradient predicts shifts in plant community response to climate change in alpine meadow on the Tibetan plateau. Sci. Total Environ..

[51] Zhang J.H., Huang Y.M. (2016). Biodiversity and stability mechanisms: Understanding and future research. Acta Ecol. Sin..

[52] Hao M., von Gadow K., Alavi S.J., Álvarez-González J.G., Baluarte-Vásquez J.R., Corral-Rivas J., Hui G., Korol M., Kumar R., Liang J. (2021). A classification of woody communities based on biological dissimilarity. Appl. Veg. Sci..

[53] Dong L.S., Zhang X.D., Zhou J.X., Song A.Y. (2007). Quantitative classification and ordination of shrub species and communities in a loess landscape of western Shanxi. Acta Ecol. Sin..

[54] Liu Q.F., Kang M.Y., Liu Q.R. (2006). Quantitative classification and environmental interpretation of forest tree species in hungou, zhongtiao mountain. Chin. J. Plant Ecol..

[55] Chen Y., Wang H.L., Han J.W., Wei B.L., Jia H.R., Ye Y.Z., Yuan Z.L. (2014). Numerical classification, ordination and species diversity along elevation gradients of the forest community in Xiaoqinling. Acta Ecol. Sin..

[56] He H.Q., Li S.C., Sun H.L., Liu S.C., Xiong W.L. (2008). Quantitative classification and ordination of Jinping hydropower station, Sichuan Province, China. Acta Ecol. Sin..

[57] Shi H., Xie F.L., Zhou Q., Shu X., Zhang K.R., Dang C.Q., Feng S.Y., Zhang Q.F., Dang H.S. (2019). Effects of topography on tree community structure in a Deciduous Broad-Leaved Forest in North-Central China. Forests.

[58] Shahriari H., Vajari K.A., Pilehvar B., Heydari M. (2020). Diversity and biomass of different functional groups of herbaceous species along an altitudinal gradient in the semi-arid Zagros mountain forests of Iran. J. For. Res..

[59] Korner C. (2007). The use of ‘altitude’ in ecological research. Trends Ecol. Evol..

[60] Rumpf S.B., Hulber K., Klonner G., Moser D., Schutz M., Wessely J., Willner W., Zimmermann N.E., Dullinger S. (2018). Range dynamics of mountain plants decrease with elevation. Proc. Natl. Acad. Sci. USA.

[61] Li S.Q., Liber K. (2018). Influence of different revegetation choices on plant community and soil development nine years after initial planting on a reclaimed coal gob pile in the Shanxi mining area, China. Sci. Total Environ..

[62] Cao J.J., Wang X.Y., Adamowski J.F., Biswas A., Liu C.F., Chang Z.Q., Feng Q. (2020). Response of leaf stoichiometry of *Oxytropis ochrocephala* to elevation and slope aspect. Catena.

[63] Qin Y.Y., Adamowski J.F., Deo R.C., Hu Z.Y., Cao J.J., Zhu M., Feng Q. (2019). Controlling factors of plant community composition with respect to the slope aspect gradient in the Qilian Mountains. Ecosphere.

[64] Qin Y.Y., Feng Q., Adamowski J.F., Zhu M., Zhang X.F. (2021). Community level response of leaf stoichiometry to slope aspect in a montane environment: A case study from the Central Qilian Mountains, China. Glob. Ecol. Conserv..

[65] Cao F.Q., Dan L., Ma Z.G., Gao T. (2020). Assessing the regional climate impact on terrestrial ecosystem over East Asia using coupled models with land use and land cover forcing during 1980–2010. Sci. Rep..

[66] Shen Z.H., Zhang X.S. (2000). A quantitative analysis on the floristic elements of the Chinese subtropical region and their spatial patterns. J. Syst. Evol..

[67] Perrino E.V., Tomaselli V., Wagensommer R.P., Silletti G.N., Esposito A., Stinca A. (2022). *Ophioglossum lusitanicum* L.: New Records of Plant Community and 92/43/EEC Habitat in Italy. Agronomy.

[68] Perrino E.V., Mahmoud Z.N.A., Valerio F., Tomaselli V., Wagensommer R.P., Trani A. (2023). Synecology of *Lagoecia cuminoides* L. in Italy and evaluation of functional compounds presence in its water or hydroalcoholic extracts. Sci. Rep..

[69] Yan P.X., Hou H., Lv Y.Z., Zhang H.Y., Li J., Shao L.L., Xie Q.M., Liang Y.L., Li J.Y., Ni X.L. (2023). Diversity characteristics of arbuscular mycorrhizal fungi communities in the soil along successional altitudes of Helan Mountain, arid, and semi-arid regions of China. Front. Microbiol..

[70] Yang Y., Qiu K.Y., Xie Y.Z., Li X.C., Zhang S., Liu W.S., Huang Y.Y., Cui L.Y., Wang S.Y., Bao P.G. (2023). Geographical, climatic, and soil factors control the altitudinal pattern of rhizosphere microbial diversity and its driving effect on root zone soil multifunctionality in mountain ecosystems. Sci. Total Environ..

[71] Wu M.Y., Chen L., Ma J.P., Zhang Y.Q., Li X.B., Pang D.B. (2023). Aggregate-associated carbon contributes to soil organic carbon accumulation along the elevation gradient of Helan Mountains. Soil Biol. Biochem..

[72] Ahmad M., Uniyal S.K., Batish D., Singh H.P., Jaryan V., Kohli R.K. (2020). Patterns of plant communities along vertical gradient in dhauladhar mountains in lesser Himalayas in north-Western India. Sci. Total Environ..

[73] Fick S.E., Hijmans R.J. (2017). WorldClim 2: New 1-km spatial resolution climate surfaces for global land areas. Int. J. Climatol..

[74] Ni J. (2017). An introduction to bioclimatic factors in global change research. Quat. Sci..

[75] Zhang J.T., Zhang M., Mian R. (2016). Effects of elevation and disturbance gradients on forest diversity in the Wulingshan Nature Reserve, North China. Environ. Earth Sci..

[76] Yang Q.C., Zhang H.H., Wang L.H., Ling F., Wang Z.X., Li T.T., Huang J.L. (2021). Topography and soil content contribute to plant community composition and structure in subtropical evergreen-deciduous broadleaved mixed forests. Plant Divers..

[77] Liu R.H., Tu H.R., Li J.F., Liang S.C., Jiang Y., Rong C.Y., Li Y.J. (2019). Numerical classification and ordination of *Cyclobalanopsis glauca* communities in karst hills of Guilin, Southwest China. Acta Ecol. Sin..

[78] Tian Y., Zhao Z.W., Liu Y. (2022). Classification and ordination of bryophyte communities in alpine meadow of eastern Tibet. Acta Ecol. Sin..

[79] Gholizadeh H., Naqinezhad A., Chytry M. (2020). Classification of the Hyrcanian forest vegetation, Northern Iran. Appl. Veg. Sci..

[80] Haq F., Ahmad H., Iqbal Z., Alam M., Aksoy A. (2017). Multivariate approach to the classification and ordination of the forest ecosystem of Nandiar valley western Himalayas. Ecol. Indic..

