# Supplementary material for: Diversity Patterns of Plant Communities along an Elevational Gradient in Arid and Semi-Arid Mountain Ecosystems in China
Source: Plants (Basel). 2024 Oct 12;13(20):2858. doi: 10.3390/plants13202858 (PMC11511201; doi:10.3390/plants13202858)
Supplement: Supplementary file 1 [file plants-13-02858-s001.zip › plants-3210542-supplementary.pdf]

plants

Article

Diversity Patterns of plant communities along an elevational gradient in arid and semi-arid mountain ecosystems in China

Xinrui He [1], Fan Yin [1], Muhammad Arif [1], Jie Zheng [1,2], Yangyi Chen [1], Qianwen Geng [1], Xilu Ni [3], Changxiao Li [1,2,*]

1 Key Laboratory of Eco-environments in Three Gorges Reservoir Region (Ministry of Education), Chongqing Key Laboratory of Plant Ecology and Resources Research in Three Gorges Reservoir Region, School of Life Sciences, Southwest University, Chongqing 400715, China; hxr2018@email.swu.edu.cn (X. H.), yinfan0801@email.swu.edu.cn (F. Y.), muhammadarif@swu.edu.cn (M. A.), jiezheng@email.swu.edu.cn (J. Z.), cyy372771162@email.swu.edu.cn (Y. C.), gqw0725@email.swu.edu.cn (Q. G.), nixilu110@163.com (X. N.), lichangx@swu.edu.cn (C. L.).

2 Biological Science Research Center, Academy for Advanced Interdisciplinary Studies, Southwest University, Chongqing, 400715, China.

3 Breeding Base for State Key Laboratory of Land Degradation and Ecological Restoration in Northwest China, College of Ecology and Environment, Ningxia University, Yinchuan, 750021, China.

* Correspondence: lichangx@swu.edu.cn (C.L.); Tel.: +86-23-68253616

Table S1. Plant species identified from the study area.

S. NO.	Family	Plant species
1	Cupressaceae	*Juniperus rigida* Siebold & Zucc.
2	Cupressaceae	*Juniperus sabina* L.
3	Pinaceae	*Picea asperata* Mast.
4	Pinaceae	*Picea crassifolia* Kom
5	Pinaceae	*Pinus tabuliformis* Carrière
6	Apiaceae	*Bupleurum chinense* Franch.
7	Apiaceae	*Bupleurum smithii* var. *parvifolium* R. H. Shan & Yin Li
8	Apiaceae	*Seseli intramongolicum* Ma
9	Asclepiadaceae	*Cynanchum thesioides* (Freyn) K. Schum.
10	Asteraceae	*Ajania fruticulosa* (Ledeb.) Poljakov
11	Asteraceae	*Artemisia dubia* Wall. ex Besser subf. intermedia Pamp
12	Asteraceae	*Artemisia mongolica* (Fisch. ex Besser) Nakai
13	Asteraceae	*Artemisia scoparia* Waldst. & Kit.
14	Asteraceae	*Aster hispidus* Thunb.
15	Asteraceae	*Chrysanthemum chanetii* H. Lév.
16	Asteraceae	*Chrysanthemum indicum* L.
17	Asteraceae	*Crepis rigescens* Diels
18	Asteraceae	*Echinops przewalskyi* Iljin
19	Asteraceae	*Leontopodium leontopodioides* (Willd.) Beauverd
20	Asteraceae	*Lipschitzia divaricata* (Turcz.) Zaika, Sukhor. & N. Kilian
21	Asteraceae	*Scorzonera sinensis* Lipsch. & Krasch. ex Lipsch.
22	Asteraceae	*Synotis atractylidifolia* (Y. Ling) C. Jeffrey & Y. L. Chen
23	Asteraceae	*Takhtajaniantha austriaca* (Willd.) Zaika, Sukhor. & N. Kilian
24	Asteraceae	*Taraxacum mongolicum* Hand.-Mazz.
25	Asteraceae	*Tugarinovia mongolica* Iljin
26	Berberidaceae	*Berberis dubia* C. K. Schneid.
27	Boraginaceae	*Arnebia guttata* Bunge
28	Boraginaceae	*Microula sikkimensis* (C. B. Clarke) Hemsl.
29	Brassicaceae	*Braya humilis* (C. A. Mey.) B. L. Rob.
30	Brassicaceae	*Draba eriopoda* Turcz. ex Ledeb.
31	Brassicaceae	*Sterigmostemum matthioloides* (Franch.) Botsch.
32	Caprifoliaceae	*Lonicera microphylla* Willd. ex Roem. & Schult.
33	Caryophyllaceae	*Dianthus superbus* L.
34	Caryophyllaceae	*Eremogone formosa* (Fisch. ex Ser.) Fenzl
35	Caryophyllaceae	*Silene gallica* L.
36	Chenopodiaceae	*Anabasis brevifolia* C. A. Mey.
37	Chenopodiaceae	*Caroxylon passerinum* (Bunge) Akhani & Roalson
38	Chenopodiaceae	*Chenopodium iljinii* Golosk.
39	Chenopodiaceae	*Krascheninnikovia ceratoides* (L.) Gueldenst.
40	Chenopodiaceae	*Oreosalsola laricifolia* (Litv. ex Drobow) Akhani
41	Chenopodiaceae	*Oxybasis glauca* (L.) S. Fuentes, Uotila & Borsch
42	Chenopodiaceae	*Salsola collina* Pall.
43	Convolvulaceae	*Convolvulus tragacanthoides* Turcz.
44	Crassulaceae	*Orostachys fimbriata* (Turcz.) A. Berger
45	Cyperaceae	*Carex kansuensis* Nelmes
46	Ephedraceae	*Ephedra rhytidosperma* Pachom.
47	Fabaceae	*Astragalus ellipsoideus* auct. Non Ledeb. : Y. X. Liou
48	Fabaceae	*Astragalus membranaceus* var. *mongholicus* (Bunge) P. K. Hsiao
49	Fabaceae	*Caragana brachypoda* Pojark.

50	Fabaceae	*Caragana korshinskii* Kom.
51	Fabaceae	*Caragana roborovskyi* Kom.
52	Fabaceae	*Caragana stenophylla* Pojark.
53	Fabaceae	*Hedysarum petrovii* Yakovlev
54	Fabaceae	*Lespedeza davurica* auct. non (Laxm.) Schindl. : V. N. Vassil.
55	Fabaceae	*Oxytropis aciphylla* Ledeb.
56	Fabaceae	*Thermopsis lanceolata* R. Br.
57	Gentianaceae	*Gentiana macrophylla* Pall.
58	Geraniaceae	*Erodium stephanianum* Willd.
59	Iridaceae	*Iris loczyi* Kanitz
60	Lamiaceae	*Lagochilus ilicifolius* Bunge
61	Lamiaceae	*Phlomoides mongolica* (Turcz.) Kamelin & A. L. Budantzev
62	Liliaceae	*Allium yanchiense* J. M. Xu
63	Liliaceae	*Polygonatum odoratum* (Mill.) Druce
64	Liliaceae	*Polygonatum sibiricum* Redouté
65	Loganiaceae	*Buddleja alternifolia* Maxim.
66	Oleaceae	*Syringa oblata* Lindl.
67	Plantaginaceae	*Plantago asiatica* L.
68	Plantaginaceae	*Plantago depressa* Willd
69	Plumbaginaceae	*Limonium tenellum* (Turcz.) Kuntze
70	Poaceae	*Achnatherum saposhnikovii* (Roshev.) Nevski
71	Poaceae	*Agropyron cristatum* (L.) Gaertn.
72	Poaceae	*Agropyron mongolicum* Keng
73	Poaceae	*Bromus japonicus* Thunb.
74	Poaceae	*Cleistogenes squarrosa* (Trin.) Keng
75	Poaceae	*Leymus chinensis* (Trin. ex Bunge) Tzvelev
76	Poaceae	*Melica scabrosa* Trin.
77	Poaceae	*Neotrinia splendens* (Trin.) M. Nobis, P. D. Gudkova & A. Nowak
78	Poaceae	*Poa annua* L.
79	Poaceae	*Ptilagrostis pelliotii* (Danguy) Grubov
80	Poaceae	*Stipa przewalskyi* Roshev.
81	Poaceae	*Stipa tianschanica* var. *gobica* (Roshev.) P. C. Kuo & Y. H. Sun
82	Polygalaceae	*Polygala tenuifolia* Willd.
83	Primulaceae	*Androsace mariae* Kanitz
84	Primulaceae	*Androsace umbellata* (Lour.) Merr.
85	Ranunculaceae	*Clematis aethusifolia* Turcz.
86	Ranunculaceae	*Clematis brevicaudata* DC.
87	Ranunculaceae	*Clematis florida* Thunb.
88	Ranunculaceae	*Clematis fruticosa* Turcz.
89	Ranunculaceae	*Clematis macropetala* Ledeb.
90	Ranunculaceae	*Delphinium grandiflorum* L.
91	Ranunculaceae	*Thalictrum aquilegiifolium* var. *sibiricum* Regel & Tiling
92	Rhamnaceae	*Rhamnus maximovicziana* J. J. Vassil.
93	Rhamnaceae	*Ziziphus jujuba* var. spinosa (Bunge) Hu ex H. F. Chow
94	Rosaceae	*Cotoneaster zabelii* C. K. Schneid.
95	Rosaceae	*Dasiphora fruticosa* (L.) Rydb.
96	Rosaceae	*Dasiphora parvifolia* (Fisch. ex Lehm.) Juz.
97	Rosaceae	*Potentilla glabra* var. *veitchii* (Wils.) Hand.-Mazz.
98	Rosaceae	*Potentilla multifida* L.
99	Rosaceae	*Potentilla sischanensis* Bunge ex Lehm.
100	Rosaceae	*Prunus mongolica* Maxim.

101	Rosaceae	*Rosa xanthina* Lindl.
102	Rosaceae	*Sanguisorba alpina* Bunge
103	Rosaceae	*Sibbaldianthe adpressa* (Bunge) Juz.
104	Rosaceae	*Sibbaldianthe bifurca* (L.) Kurtto & T. Erikss.
105	Rosaceae	*Spiraea lasiocarpa* Kar. & Kir.
106	Rosaceae	*Spiraea trilobata* L.
107	Rubiaceae	*Galium odoratum* (L.) Scop.
108	Rubiaceae	*Leptodermis ordosica* H. C. Fu & E. W. Ma
109	Rubiaceae	*Rubia cordifolia* L.
110	Salicaceae	*Populus davidiana* Dode
111	Saxifragaceae	*Ribes pulchellum* var. *manshuriense*
112	Scrophulariaceae	*Cymbaria mongolica* Maxim.
113	Scrophulariaceae	*Rehmannia glutinosa* (Gaertn.) Libosch. ex Fisch. & C. A. Mey.
114	Tamaricaceae	*Reaumuria songarica* (Pall.) Maxim.
115	Tamaricaceae	*Reaumuria trigyna* Maxim.
116	Ulmaceae	*Ulmus glaucescens* Franch.
117	Urticaceae	*Urtica cannabina* L.
118	Verbenaceae	*Caryopteris mongholica* Bunge
119	Violaceae	*Viola arcuata* Blume
120	Zygophyllaceae	*Peganum harmala* L.
121	Zygophyllaceae	*Zygophyllum xanthoxylum* (Bunge) Maxim.

Note: the nomenclature and species delimitation were conducted using relevant methods as references [1,2].

Table S2. Frequency statistics of the occurrence of each species among the 23 sampling sites.

Layer	Plant species	Number of sampling sites with the species present
Arbor	*Juniperus rigida* Siebold & Zucc.	5
Arbor	*Picea asperata* Mast.	1
Arbor	*Picea crassifolia* Kom	1
Arbor	*Pinus tabuliformis* Carrière	3
Arbor	*Populus davidiana* Dode	1
Arbor	*Ulmus glaucescens* Franch.	4
Shrub	*Juniperus sabina* L.	1
Shrub	*Anabasis brevifolia* C. A. Mey.	1
Shrub	*Berberis dubia* C. K. Schneid.	5
Shrub	*Buddleja alternifolia* Maxim.	2
Shrub	*Caragana brachypoda* Pojark.	6
Shrub	*Caragana korshinskii* Kom.	1
Shrub	*Caragana roborovskyi* Kom.	3
Shrub	*Caragana stenophylla* Pojark.	8
Shrub	*Caroxylon passerinum* (Bunge) Akhani & Roalson	1
Shrub	*Caryopteris mongholica* Bunge	3
Shrub	*Clematis brevicaudata* DC.	1
Shrub	*Clematis fruticosa* Turcz.	1
Shrub	*Convolvulus tragacanthoides* Turcz.	11
Shrub	*Cotoneaster zabelii* C. K. Schneid.	7
Shrub	*Dasiphora fruticosa* (L.) Rydb.	4
Shrub	*Dasiphora parvifolia* (Fisch. ex Lehm.) Juz.	10
Shrub	*Ephedra rhytidosperma* Pachom.	5
Shrub	*Krascheninnikovia ceratoides* (L.) Gueldenst.	1
Shrub	*Leptodermis ordosica* H. C. Fu & E. W. Ma	8
Shrub	*Lonicera microphylla* Willd. ex Roem. & Schult.	6
Shrub	*Oreosalsola laricifolia* (Litv. ex Drobow) Akhani	11
Shrub	*Oxytropis aciphylla* Ledeb.	9
Shrub	*Potentilla glabra* var. *veitchii* (Wils.) Hand.-Mazz.	4
Shrub	*Prunus mongolica* Maxim.	10
Shrub	*Reaumuria trigyna* Maxim.	1
Shrub	*Reaumuria songarica* (Pall.) Maxim.	1
Shrub	*Rhamnus maximovicziana* J. J. Vassil.	6
Shrub	*Ribes pulchellum* var. *manshuriense*	3
Shrub	*Rosa xanthina* Lindl.	7
Shrub	*Spiraea lasiocarpa* Kar. & Kir.	8
Shrub	*Spiraea trilobata* L.	1
Shrub	*Syringa oblata* Lindl.	1
Shrub	*Ulmus glaucescens* Franch.	7
Shrub	*Ziziphus jujuba* var. *spinosa* (Bunge) Hu ex H. F. Chow	7
Shrub	*Zygophyllum xanthoxylum* (Bunge) Maxim.	2
Herb	*Achnatherum saposhnikovii* (Roshev.) Nevski	2
Herb	*Agropyron cristatum* (L.) Gaertn.	2
Herb	*Agropyron mongolicum* Keng	8
Herb	*Ajania fruticulosa* (Ledeb.) Poljakov	11
Herb	*Allium yanchiense* J. M. Xu	1
Herb	*Androsace mariae* Kanitz	1
Herb	*Androsace umbellata* (Lour.) Merr.	1
Herb	*Arnebia guttata* Bunge	1

Herb	*Artemisia dubia* Wall. ex Besser subf. intermedia Pamp	5
Herb	*Artemisia mongolica* (Fisch. ex Besser) Nakai	5
Herb	*Artemisia scoparia* Waldst. & Kit.	2
Herb	*Aster hispidus* Thunb.	5
Herb	*Astragalus ellipsoideus* auct. Non Ledeb. : Y. X. Liou	4
Herb	*Astragalus membranaceus* var. *mongholicus* (Bunge) P. K. Hsiao	2
Herb	*Braya humilis* (C. A. Mey.) B. L. Rob.	5
Herb	*Bromus japonicus* Thunb.	2
Herb	*Bupleurum chinense* Franch.	1
Herb	*Bupleurum smithii* var. *parvifolium* R. H. Shan & Yin Li	2
Herb	*Carex kansuensis* Nelmes	7
Herb	*Chenopodium iljinii* Golosk.	1
Herb	*Chrysanthemum chanetii* H. Lév.	1
Herb	*Chrysanthemum indicum* L.	2
Herb	*Cleistogenes squarrosa* (Trin.) Keng	10
Herb	*Clematis aethusifolia* Turcz.	4
Herb	*Clematis florida* Thunb.	1
Herb	*Clematis macropetala* Ledeb.	1
Herb	*Crepis rigescens* Diels	1
Herb	*Cymbaria mongolica* Maxim.	4
Herb	*Cynanchum thesioides* (Freyn) K. Schum.	2
Herb	*Delphinium grandiflorum* L.	1
Herb	*Dianthus superbus* L.	1
Herb	*Draba eriopoda* Turcz. ex Ledeb.	1
Herb	*Echinops przewalskyi* Iljin	1
Herb	*Eremogone formosa* (Fisch. ex Ser.) Fenzl	1
Herb	*Erodium stephanianum* Willd.	1
Herb	*Galium odoratum* (L.) Scop.	2
Herb	*Gentiana macrophylla* Pall.	2
Herb	*Hedysarum petrovii* Yakovlev	1
Herb	*Limonium tenellum* (Turcz.) Kuntze	1
Herb	*Iris loczyi* Kanitz	4
Herb	*Lagochilus ilicifolius* Bunge	9
Herb	*Leontopodium leontopodioides* (Willd.) Beauverd	2
Herb	*Lespedeza davurica* auct. non (Laxm.) Schindl. : V. N. Vassil.	9
Herb	*Leymus chinensis* (Trin. ex Bunge) Tzvelev	2
Herb	*Lipschitzia divaricata* (Turcz.) Zaika, Sukhor. & N. Kilian	1
Herb	*Melica scabrosa* Trin.	1
Herb	*Microula sikkimensis* (C. B. Clarke) Hemsl.	2
Herb	*Neotrinia splendens* (Trin.) M. Nobis, P. D. Gudkova & A. Nowak	4
Herb	*Orostachys fimbriata* (Turcz.) A. Berger	1
Herb	*Oxybasis glauca* (L.) S. Fuentes, Uotila & Borsch	1
Herb	*Peganum harmala* L.	1
Herb	*Phlomoides mongolica* (Turcz.) Kamelin & A. L. Budantzev	1
Herb	*Plantago asiatica* L.	1
Herb	*Plantago depressa* Willd	1
Herb	*Poa annua* L.	2
Herb	*Polygala tenuifolia* Willd.	6
Herb	*Polygonatum odoratum* (Mill.) Druce	3
Herb	*Polygonatum sibiricum* Redouté	1
Herb	*Potentilla multifida* L.	2

Herb	*Potentilla sischanensis* Bunge ex Lehm.	5
Herb	*Ptilagrostis pelliotii* (Danguy) Grubov	4
Herb	*Reaumuria trigyna* Maxim.	2
Herb	Rubia cordifolia L.	1
Herb	*Salsola collina* Pall.	1
Herb	*Sanguisorba alpina* Bunge	1
Herb	*Scorzonera sinensis* Lipsch. & Krasch. ex Lipsch.	1
Herb	*Seseli intramongolicum* Ma	1
Herb	*Sibbaldianthe adpressa* (Bunge) Juz.	1
Herb	*Sibbaldianthe bifurca* (L.) Kurtto & T. Erikss.	2
Herb	*Silene gallica* L.	1
Herb	*Sterigmostemum matthioloides* (Franch.) Botsch.	1
Herb	*Stipa przewalskyi* Roshev.	13
Herb	*Stipa tianschanica* var. *gobica* (Roshev.) P. C. Kuo & Y. H. Sun	10
Herb	*Synotis atractylidifolia* (Y. Ling) C. Jeffrey & Y. L. Chen	2
Herb	*Takhtajaniantha austriaca* (Willd.) Zaika, Sukhor. & N. Kilian	1
Herb	*Taraxacum mongolicum* Hand.-Mazz.	3
Herb	*Thalictrum aquilegiifolium* var. sibiricum	4
Herb	*Thermopsis lanceolata* R. Br.	2
Herb	*Tugarinovia mongolica* Iljin	1
Herb	*Urtica cannabina* L.	1
Herb	*Viola arcuata* Blume	1

References

1. Turland, N. J.; Wiersema, J. H.; Barrie, F.R.; Greuter, W.; Hawksworth, D. L.; Herendeen, P.S.; Knapp, S.; Kusber, W.H.; Li, D.Z.; Marhold, K.; May, T.W.; McNeill, J.; Monro, A.M.; Prado, J.; Price M.J.; Smith, G. International Code of Nomenclature for algae, fungi, and plants (Shenzhen Code). 2018. (Regnum Vegetabile, 159). XXXVIII, 254 p. gr8vo. Hardcover. (ISBN 978-3-946583-16-5).
2. Rouhan, G.; Gaudeul, M. Plant Taxonomy: A Historical Perspective, Current Challenges, and Perspectives. *Methods Mol Biol*, **2021**, 2222, 1-38, doi: 10.1007/978-1-0716-0997-2_1.
